# Reviewer Acknowledgement 2014

**DOI:** 10.1186/1868-7083-6-3

**Published:** 2014-01-28

**Authors:** Ulrich Mahlknecht

**Affiliations:** 1 Department for Internal Medicine - Hematology/Oncology, St. Lukas Klinik, Solingen, Germany; 2 Department of Internal Medicine, Division of Immunotherapy and Gene Therapy, Saarland University Medical Center, Homburg/Saar, Germany

## Abstract

**Contributing reviewers:**

The Editors of Clinical Epigenetics would like to thank all our reviewers who have contributed to the journal in volume 5 (2013).

## 

Jorge Alegría

Mexico

Anne-Marie Baird

Ireland

Nafisa Huseni Balasinor

India

Maria Berdasco

Spain

Agnes Berendsen

United States of America

Gerhard Binder

Germany

Richard Bucala

United States of America

Alan Burnett

United Kingdom

Hyang-Min Byun

United States of America

Murray Cairns

Australia

Kevin Caldwell

United States of America

Sridar Chittur

United States of America

Ramen Chmait

United States of America

James Close

United Kingdom

Debora Costa

Brazil

Aldona Dembinska-Kiec

Poland

Thomas Eggermann

Germany

Osman El-Maarri

Germany

Stephen Fava

Malta

Michael Fry

Israel

Rebecca Fry

United States of America

Tod Fullston

Australia

Carlo Gaetano

Germany

David Gavin

United States of America

Anita Göndör

Sweden

Helen Gunter

Germany

Markus Herrmann

Italy

Eric Hervouet

France

Luke Hesson

Australia

Michael Holick

United States of America

Tomislav Horvat

Croatia

Tinghui Hu

United States of America

Yanling Hu

China

Hailiang Huang

United States of America

Henry Kahn

United States of America

Maryse Lemaire

Canada

Amy Lossie

United States of America

Robyn Lucas

Australia

Hendrik Marks

Netherlands

Lee Martin

United States of America

Saverio Minucci

Italy

Murray Mitchell

Australia

Richard L Momparler

Canada

Eva Morales

Spain

Alma Nauta

Netherlands

Ruta Navakauskiene

Lithuania

Clara Nervi

Italy

Boris Novakovic

Australia

Longjuan Qin

China

Rocio Melissa Rivera

United States of America

Tina Rönn

Sweden

Tessa Roseboom

Netherlands

Richard Saffery

Australia

Gerry Schwalfenberg

Canada

Karen Seiter

United States of America

Yoel Shufaro

Israel

Adrian Stankiewicz

Poland

Pao-Ling Torng

Taiwan

Gloria Valdes

Chile

Carine Van Lint

Belgium

José M. Villalba

Spain

Anders Waage

Norway

Maria Wing

United States of America

Stefan Wölfl

Germany

Yu Mi Woo

South Korea

Hua Xu

China

Ian Yang

Australia

Min You

United States of America

Jinying Zhao

United States of America

Vlatka Zoldos

Croatia

